# Association between daily breakfast habit during pregnancy and neurodevelopment in 3-year-old offspring: The Japan Environment and Children’s Study

**DOI:** 10.1038/s41598-024-55912-x

**Published:** 2024-03-15

**Authors:** Karin Imaizumi, Tsuyoshi Murata, Hirotaka Isogami, Toma Fukuda, Hyo Kyozuka, Shun Yasuda, Akiko Yamaguchi, Akiko Sato, Yuka Ogata, Kosei Shinoki, Mitsuaki Hosoya, Seiji Yasumura, Koichi Hashimoto, Keiya Fujimori, Hidekazu Nishigori, The Japan Environment and Children’s Study (JECS) Group, Michihiro Kamijima, Shin Yamazaki, Yukihiro Ohya, Reiko Kishi, Nobuo Yaegashi, Chisato Mori, Shuichi Ito, Zentaro Yamagata, Hidekuni Inadera, Takeo Nakayama, Tomotaka Sobue, Masayuki Shima, Hiroshige Nakamura, Narufumi Suganuma, Koichi Kusuhara, Takahiko Katoh

**Affiliations:** 1 Fukushima Regional Center for the Japan Environment and Children’s Study, 1 Hikarigaoka, Fukushima, 960-1295 Japan; 2 Department of Obstetrics and Gynecology, Fukushima Medical University School of Medicine, 1 Hikarigaoka, Fukushima, 960-1295 Japan; 3 Department of Pediatrics, Fukushima Medical University School of Medicine, 1 Hikarigaoka, Fukushima, 960-1295 Japan; 4 Department of Public Health, Fukushima Medical University School of Medicine, 1 Hikarigaoka, Fukushima, 960-1295 Japan; 5 Fukushima Medical Center for Children and Women, Fukushima Medical University, 1 Hikarigaoka, Fukushima, 960-1295 Japan; 6 Nagoya City University, Nagoya, Japan; 7 National Institute for Environmental Studies, Tsukuba, Japan; 8 National Center for Child Health and Development, Tokyo, Japan; 9 Hokkaido University, Sapporo, Japan; 10 Tohoku University, Sendai, Japan; 11 Chiba University, Chiba, Japan; 12 Yokohama City University, Yokohama, Japan; 13 University of Yamanashi, Chuo, Japan; 14 University of Toyama, Toyama, Japan; 15 Kyoto University, Kyoto, Japan; 16 Osaka University, Suita, Japan; 17 Hyogo Medical University, Nishinomiya, Japan; 18 Tottori University, Yonago, Japan; 19 Kochi University, Nankoku, Japan; 20 University of Occupational and Environmental Health, Kitakyushu, Japan; 21 Kumamoto University, Kumamoto, Japan

**Keywords:** Medical research, Epidemiology

## Abstract

The association between daily breakfast habits during pregnancy and offspring neurodevelopment remains unknown. We evaluated the association between breakfast habits during pregnancy and offspring neurodevelopment. Data of 72,260 women with singleton deliveries at and after 37 weeks of gestation enrolled during 2011–2014 in the Japan Environment and Children’s Study were analysed. Offspring neurodevelopmental delays at 3 years of age were evaluated using the Ages and Stages Questionnaire, Third Edition (ASQ-3). Participants were stratified by tertiles of maternal daily energy intake (DEI) (Groups 1, 2, and 3:< 1400, 1400–1799, and ≥ 1800 kcal, respectively) during pregnancy and by offspring sex. The adjusted odds ratio (aOR) for abnormality in communication among participants with daily breakfast consumption habit was 0.87 (95% confidence interval, 0.80–0.96). A stratified analysis based on total DEI showed no significant differences in the neurodevelopment of Group 1 offspring. The aOR for abnormality in communication was 0.80 (95% confidence interval, 0.68–0.94) in Group 2. The aOR for abnormality in personal–social characteristics was 0.84 (95% confidence interval, 0.71–0.99) in Group 3. Maternal daily breakfast habits are associated with offspring neurodevelopment at 3 years of age, with the association influenced by maternal DEI and offspring sex.

## Introduction

The prevalence of children diagnosed with mental, behavioural, and neurodevelopmental disorders has significantly increased, with approximately 15% of children aged 2–8 years having one or more neurodevelopmental disorder^1^. Infection and inflammatory stress, quality of care, and nutrition affect neurodevelopment in children^2^. Understanding factors that contribute to neurodevelopmental delays is critical to find solutions to reduce the prevalence of neurodevelopmental disorders.

Given that childhood neurodevelopmental delays typically manifest early in development^3^, the role of maternal nutritional factors in foetal development has emerged as an important research topic in the twentieth century^4^. Periconceptional and prenatal environments are critical for foetal brain development^4^. Exposing the developing brain to nutritional deficiencies impairs myelination and interferes with cognitive function and other neurodevelopmental processes^5^. Moreover, nutrient deficiencies affect cell proliferation in foetal brain regions in early pregnancy and synaptogenesis and dendritic arborisation in neuronal neurons in late pregnancy^6^. Thus, maternal nutritional deficiencies can cause cognitive and behavioural deficits with permanent brain dysfunction in the foetus and structural changes in developing and even adult brains^7^. The intrauterine environment affected by maternal nutrition is, therefore, important because it can have irreversible effects on the child’s future brain development.

Antonow-Schlorke et al. reported that in baboons, which are human-like primates with many anatomic, physiologic, and genetic similarities to humans^8^, maternal nutritional deficiencies caused impairments in foetal cerebral development, including of glial maturation and neuronal cell formation^9^. It has also been reported that in humans, maternal undernutrition leads to an increased risk of schizophrenia in the offspring^10^ and is associated with cognitive decline, inattention, and behavioural abnormalities^11^. The World Health Organization (WHO) has guidelines for antenatal care but does not detail nutritional needs of women throughout reproduction -preconception, pregnancy and lactation^12^. Therefore, there is currently no comprehensive indicator to guide pregnant women on the appropriate diet that promotes foetal neurodevelopment.

In general, regular breakfast consumption is considered a healthy habit. A study of Japanese university students reported that a breakfast habit was positively correlated with better nutritional balance, fruit and vegetable intake, and better sleep habits than groups without such a habit. Further, it was also associated with better lifestyle habits, including lower alcohol intake and smoking rates^13^. Conversely, skipping breakfast was associated with a low quality diet and high risk of nutritional deficiencies^14^. Breakfast habits are also associated with the prevention of lifestyle diseases. For example, in a systematic review and meta-analysis conducted by Bi et al., daily breakfast intake prevented type 2 diabetes^15^. In a prospective study of adult men, those who skipped breakfast had a 27% higher risk of coronary heart disease than those who consumed breakfast daily^16^. Moreover, a daily breakfast consumption habit (DBH) is associated with improved cognitive function and academic performance in both children and adults^17–20^.

However, the effects of DBH during pregnancy on obstetrics, perinatal outcomes, and further offspring neurodevelopment are unclear. Shiraishi et al. reported that skipping breakfast during pregnancy may result in low intake of fatty acids, such as eicosapentaenoic acid (EPA) and docosahexaenoic acid (DHA), protein, and β-carotene, which are necessary for foetal growth^21^. In Japan, approximately 20–30% of pregnant women skip breakfast^22^. Thus, this study aimed to analyse the association between DBH during pregnancy and the incidence of neurodevelopmental delays in offspring at 3 years of age using data from a nationwide birth cohort study. We also investigated whether the effect of DBH during pregnancy varied with maternal daily energy intake (DEI) and offspring sex. We hypothesised (1) that maternal DBH had favourable effects on offspring neurodevelopment by providing proper total daily nutrient intake and (2) that maternal DEI and offspring sex affected the association between DBH and offspring neurodevelopment.

## Methods

### Study design and data sources

This study analysed data from the Japan Environment and Children’s Study (JECS). Briefly, the JECS is a nationwide, government-funded, prospective birth cohort study started in January 2011 to investigate the effects of environmental factors on children’s health^23,24^. The JECS is funded directly by the Ministry of the Environment, Japan and involves a collaboration among the Programme Office (National Institute for Environmental Studies), Medical Support Centre (National Center for Child Health and Development), and 15 regional centres (Hokkaido, Miyagi, Fukushima, Chiba, Kanagawa, Koshin, Toyama, Aichi, Kyoto, Osaka, Hyogo, Tottori, Kochi, Fukuoka, and South Kyushu/Okinawa)^23,24^. For inclusion in the JECS, expectant mothers had to meet the following criteria: (1) reside within the study area at the time of recruitment, with an expectation to continue residing in Japan in the foreseeable future; (2) have an expected due date between 1 August 2011 and mid 2014; and (3) have the ability to participate in the study without difficulty (i.e. ability to comprehend the Japanese language and complete a self-administered questionnaire).

There were two modes of recruitment: (1) at the time of the first prenatal examination at participating obstetric facilities and (2) at local government offices that issued a pregnancy journal (i.e. the Maternal and Child Health Handbook) to all expecting mothers in Japan before they received municipal services for pregnancy, delivery, and childcare. Pregnant women were contacted through the cooperating health care providers and/or local government offices issuing Maternal and Child Health Handbooks. Those who were willing to participate were registered. Self-administered questionnaires were completed by the women during the first and second/third trimesters. Information on demographic factors, medical history, physical and mental health, lifestyle, occupation, environmental exposures at home and in the workplace, housing conditions, and socioeconomic status was collected^23,24^.

The current analysis used data released in October 2019 (data set: jecs-ta-20190930). Participants with singleton pregnancies were included in the present study. Women with abortion, stillbirth, and missing information on exposure and outcomes were excluded from the analysis. Offspring with chromosomal abnormalities, cerebral palsy, and preterm births were also excluded.

### Exposure variables

Data on DBH during pregnancy were collected once during the second or third trimester (median, 27.0 weeks of gestation) using the self-reported food frequency questionnaire (FFQ). The FFQ is generally used to assess the nutrient and food intake of subjects in large epidemiological studies in several countries, including Japan. Its validity and validity have been previously demonstrated^25–30^. Basically, the questionnaire asks about the extent to which subjects habitually consumed in the past year, with nine frequency categories ranging from rarely eaten to more than seven times a day (more than 10 drinks per day for beverages). It consists of three portion sizes: small, 0.5; medium, 1.0; and large 1.5^31,32^. In this study, eating habits such as frequency of breakfast, frequency of eating out, and eating speed were also assessed^31,32^. Energy and nutrient intakes were calculated using a food composition table prepared for the FFQ based on the fifth edition of the Japanese Food Composition Table^32^.

The original question on breakfast consumption was ‘How often did you have breakfast during the past 12 months?’ The frequency included 6 categories: < 1 time/month, 1–3 times/month, 1–2 times/week, 3–4 times/week, 5–6 times/week, and every day. Participants with and without DBH were defined as those who ate breakfast daily and those with other frequencies of consumption, respectively.

### Main outcome measures and confounding factors

We used the Japanese translation of the Ages and Stages Questionnaire, Third Edition (ASQ-3) to assess offspring neurodevelopmental delays. The ASQ-3 is a screening tool for developmental delays in children aged 1–66 months. It is widely accepted in clinical settings and research and has high reliability and validity^33,34^. The ASQ-3 comprises five domains: communication (Com), gross motor (GM), fine motor (FM), problem solving (Prob), and personal–social characteristics (Perso). In each domain, the parent answers ‘yes’, ‘sometimes’, or ‘not yet’ for specific child behaviours with scores of 10, 5, and 0, respectively, leading to a total score of 0–60. When the total score in each domain is below the specific cut-off value, the child is considered to have a neurodevelopmental delay for that domain. The Japanese version of the ASQ-3 has been validated using adjusted cut-off scores of 29.95, 39.26, 27.91, 30.03, and 29.89 for the Com, GM, FM, Prob, and Perso domains, respectively, at 3 years of age^33^.

The following factors were used as potential confounding or predictive factors: maternal age, body mass index (BMI) before pregnancy, parity, maternal smoking status, maternal alcohol consumption status, maternal educational status, annual household income, assisted reproductive technology, maternal psychological disorder, high maternal Kessler 6 (K6) scores, maternal neurodevelopmental disorders, high maternal autism spectrum quotient (AQ), lactation status at 6 months after birth, marital status, paternal age, paternal smoking status, paternal educational status, other children in the house, nursery or kindergarten, and maternal job. There was no multicollinearity, which was judged to present under the following conditions: an association between independent variables with a correlation coefficient of r > 0.8 and/or a variance inflation factor > 10.

Maternal and paternal ages were categorised as < 20, 20–29, 30–39, and ≥ 40 years. BMI before pregnancy was categorised as < 18.5, 18.5–19.9, 20.0–22.9, 23.0–24.9, and ≥ 25.0 kg/m^2^. Parity was dichotomised as nulliparous or multiparous. The participants were requested to provide information regarding their smoking status by choosing one of the following answers: ‘currently smoking’, ‘never’, ‘previously did, but quit before realising current pregnancy’, and ‘previously did, but quit after realising current pregnancy’. Those who chose ‘currently smoking’ were included in the ‘smoking’ category, and all others were included in the ‘non-smoking’ category.

Furthermore, the participants were requested to provide information regarding their alcohol consumption status by choosing one of the following answers: ‘never drank’, ‘quit drinking before pregnancy’, ‘quit drinking during the early stages of pregnancy’, and ‘kept drinking during pregnancy’^31^. Maternal participants who ‘kept drinking during pregnancy’ were included in the drinking category, and all others were included in the non-drinking category. Maternal and paternal educational status was categorised into the following four groups according to the number of years of education completed: junior high school, < 10 years; high school, 10–12 years; professional school or university, 13–16 years; and graduate school, ≥ 17 years. Annual household income was categorised into four levels: < 2,000,000, 2,000,000–5,999,999, 6,000,000–9,999,999, and ≥ 10,000,000 JPY. Maternal K6 scores ≥ 13 obtained at the first half of pregnancy were considered high^35^.

Maternal and paternal neurodevelopmental disorders included attention-deficit hyperactivity disorder, learning disorder, and pervasive developmental disorder. AQ is a self-report questionnaire for screening normally intelligent adolescents and adults with a high prevalence of high-functioning pervasive developmental disorders; a maternal and paternal AQ ≥ 7 was considered high^36^. For each confounder, ‘no answer’ was analysed as a single item.

### Statistical analysis

The women were stratified based on DBH, and maternal, paternal, and offspring characteristics were compared. Univariable and multivariable logistic regression models were used to calculate crude odds ratios (cORs), adjusted odds ratios (aORs), and 95% confidence intervals (CIs) for abnormalities in each ASQ-3 domain for participants with DBH, with participants without DBH as the reference group. Odds ratios were adjusted for potential confounding or predictive factors. Furthermore, participants were stratified by tertiles of maternal DEI during pregnancy and by offspring sex. DEI was collected based on estimated caloric intake from the FFQ questionnaire administered during the second or third trimester of pregnancy. The DEI tertiles were as follows: Group 1, < 1400 kcal; Group 2, 1400–1799 kcal; and Group 3, ≥ 1800 kcal. All statistical analyses were performed using SPSS version 26 (IBM Corp., Armonk, NY, USA). A *P*-value of < 0.05 indicated statistical significance.

### Ethical approval

The JECS protocol was conducted according to the guidelines laid down in the Declaration of Helsinki, and all procedures involving human subjects were approved by the Ministry of the Environment’s Institutional Review Board on Epidemiological Studies (No. 100910001) and the Ethics Committees of all participating institutions. Written informed consent was obtained from all subjects.

## Results

**Figure 1:**
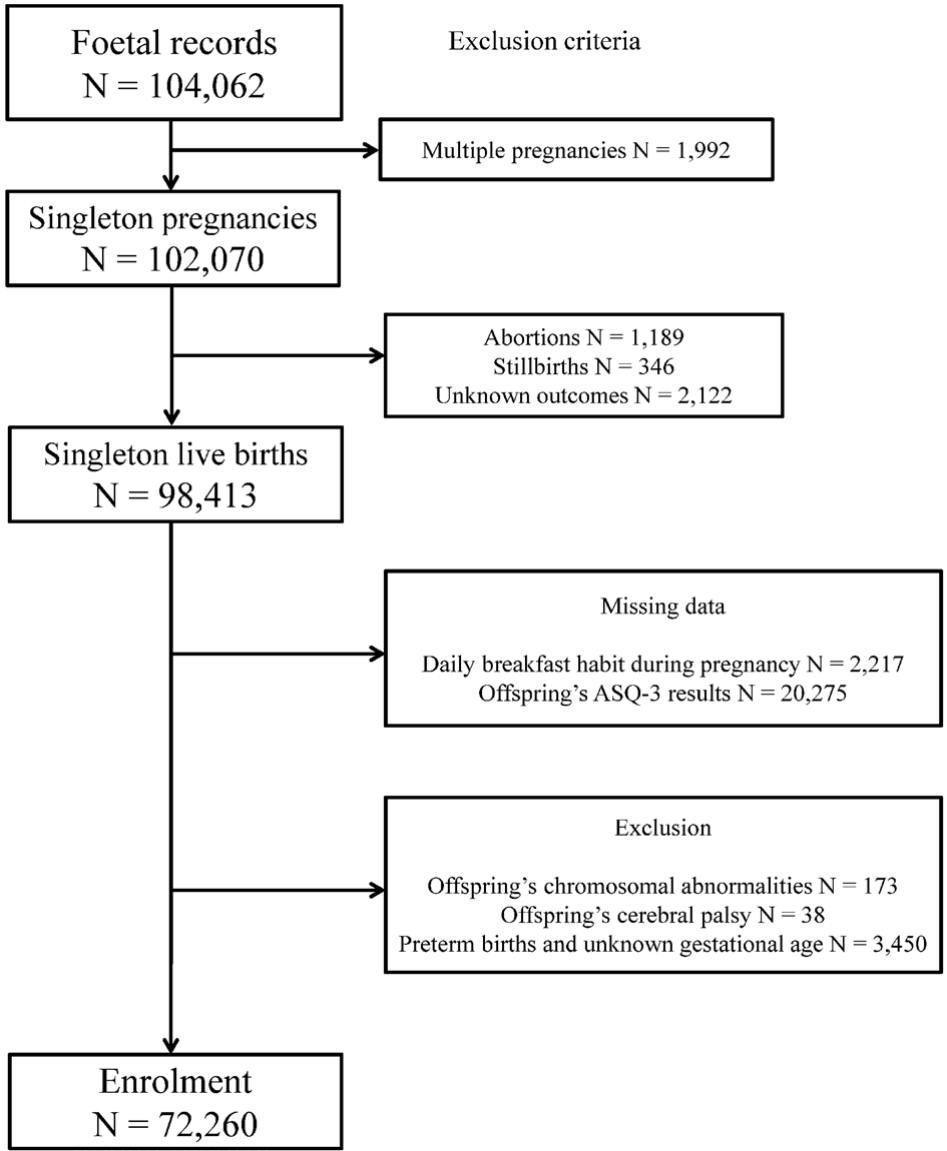
Participant enrolment flowchart.

A total of 104,062 foetal records were registered within the JECS, and 72,260 participants met the inclusion criteria (Fig. 1). The mean DEI was 1735.63 kcal/day in the overall study population and was 1774.9 kcal/day and 1626.61 kcal/day in the participants with and without DBH, respectively. Table 1 summarises the maternal, paternal, and offspring characteristics according to maternal DBH status during pregnancy. Table 2 summarises the cORs, aORs, and 95% CIs for outcomes for both offspring sexes in the participants with DBH. The aOR was 0.87 (95% CI 0.80–0.96) for abnormalities in Com among participants with DBH. No significant differences in aORs were found in GM, FM, Prob, and Perso abilities.

**Table 1:** Participant characteristics and outcomes by maternal daily breakfast consumption habit status.

	With daily breakfast consumption habit	Without daily breakfast consumption habit
Variable	N = 53,124	N = 19,136
Mean maternal daily energy intake, (standard deviation)	1774.90 kcal (747.66)	1626.61 kcal (738.39)
Maternal age (years), % (n)		
< 20	0.5 (287)	3.0 (574)
20–29	37.2 (19,739)	55.6 (10,640)
30–39	59.3 (31,524)	39.7 (7,602)
≥ 40	3.0 (1573)	1.7 (320)
No answer	0.0 (1)	0.0 (0)
BMI before pregnancy (kg/m^2^), % (n)		
< 18.5	15.9 (8,435)	16.9 (3,226)
18.5–19.9	26.0 (13,801)	24.0 (4,586)
20.0–22.9	39.0 (20,721)	36.2 (6,929)
23.0–24.9	10.4 (5,503)	10.8 (2,066)
≥ 25.0	8.7 (4,646)	12.1 (2,313)
No answer	0.0 (18)	0.1 (16)
Parity, % (n)		
Nulliparous	35.8 (19,030)	55.3 (10,582)
Multiparous	62.0 (32,962)	41.5 (7,944)
No answer	2.1 (1,132)	3.2 (610)
Maternal smoking status, % (n)		
No	97.1 (51,570)	91.2 (17,452)
Yes	2.1 (1,099)	7.6 (1,450)
No answer	0.9 (455)	1.2 (234)
Maternal alcohol consumption status, % (n)		
No	96.8 (51,407)	96.4 (18,455)
Yes	2.6 (1,383)	2.9 (562)
No answer	0.6 (334)	0.6 (119)
Maternal educational status, % (n)		
Junior high school, < 10 years	2.2 (1,184)	7.6 (1,449)
High school, 10–12 years	27.2 (14,457)	37.0 (7,089)
Professional school or university, 13–16 years	68.5 (36,366)	54.0 (10,329)
Graduate school, ≥ 17 years	1.8 (965)	1.0 (191)
No answer	0.3 (152)	0.4 (78)
Annual household income (JPY), % (n)		
< 2,000,000	3.5(1,862)	7.5 (1,434)
2,000,000–5,999,999	62.4 (33,131)	65.6 (12,555)
6,000,000–9,999,999	23.8 (12,618)	16.8 (3,206)
≥ 10,000,000	4.5 (2,411)	3.0 (572)
No answer	5.8 (3,102)	7.2 (1,369)
ART, % (n)		
No	96.1 (51,033)	97.4 (18,647)
Yes	3.5 (1,878)	2.1 (398)
No answer	0.4 (213)	0.5 (91)
Maternal psychological disorder, % (n)		
No	99.4 (52,814)	99.0 (18,944)
Yes	0.6 (310)	1.0 (192)
Maternal high K6 scores, % (n)		
No	93.3 (49,549)	92.9 (17,772)
Yes	3.5 (1,833)	3.3 (623)
No answer	3.3 (1,742)	3.9 (741)
Maternal neurodevelopmental disorders, % (n)		
No	99.6 (52,926)	99.5 (19,047)
Yes	0.0 (25)	0.1 (15)
No answer	0.3 (173)	0.4 (74)
Maternal AQ ≥ 10, % (n)		
No	91.4 (48,565)	92.0 (17,604)
Yes	2.4 (1,275)	3.1 (598)
No answer	6.2 (3,284)	4.9 (934)
Lactational status at 6 months after birth, % (n)		
Lactation only	58.4 (31,019)	49.9 (9,544)
Mixed	22.6 (11,995)	22.4 (4,295)
Formula only	14.3 (7,619)	22.2 (4,242)
No answer	4.7 (2,491)	5.5 (1055)
Marital status, % (n)		
Married	97.0 (51,542)	91.5 (17,503)
Never married	1.9 (1,022)	6.5 (1237)
Divorced	0.4 (239)	1.1 (209)
Husband died	0.0 (10)	0.0 (1)
No answer	0.6 (311)	1.0 (186)
Paternal age (years), % (n)		
< 20	0.1 (40)	0.4 (76)
20–29	13.0 (6,900)	21.3 (4,083)
30–39	32.9 (17,493)	25.6 (4,896)
≥ 40	7.2 (3,838)	5.1 (972)
No answer	46.8 (24,853)	47.6 (9,109)
Paternal smoking status, % (n)		
No	58.2 (30,906)	44.1 (8,443)
Yes	40.0 (21,244)	53.1 (10,152)
No answer	1.8 (974)	2.8 (541)
Paternal educational status, % (n)		
Junior high school, < 10 years	4.7 (2,494)	10.2 (1,949)
High school, 10–12 years	33.6 (17,869)	39.6 (7,578)
Professional school or university, 13–16 years	55.5 (29,472)	45.9 (8,787)
Graduate school, ≥ 17 years	5.5 (2,970)	3.0 (577)
No answer	0.7 (349)	1.3 (245)
Other children in house, % (n)		
No	28.2 (14,961)	41.9 (8,015)
Yes	69.7 (37,014)	54.7 (10,475)
No answer	2.2 (1,149)	3.4 (646)
Nursery or kindergarten, % (n)		
No	36.4 (19,330)	36.5 (6,987)
Yes	60.7 (32,227)	60.8 (11,631)
No answer	2.9 (1,567)	2.7 (518)
Maternal job, % (n)		
No	50.4 (26,783)	52.7 (10,083)
Yes	45.5 (24,189)	42.0 (8,032)
No answer	4.1 (2,152)	5.3 (1,021)
Offspring neurodevelopmental delays, % (n)		
Communication	3.5 (1,874)	4.3 (824)
Gross motor	4.2 (2,251)	4.1 (777)
Fine motor	7.0 (3,737)	7.9 (1,509)
Problem solving	6.9 (3,662)	7.5 (1,434)
Personal–social	2.9 (1,554)	3.4 (654)

*DEI* daily energy intake, *BMI* body mass index, *JPY* Japanese yen, *ART* assisted reproductive technology, *K6* Kessler 6, *AQ* autism spectrum quotient.

**Table 2:** Crude and adjusted ORs for abnormalities in the ASQ-3 in participants with a daily breakfast consumption habit.

Com		GM		FM		Prob		Perso	
cOR	aOR	cOR	aOR	cOR	aOR	cOR	aOR	cOR	aOR
**0.81** **(0.75–0.88)**	**0.87** **(0.80–0.96)**	1.05 (0.96–1.14)	1.07 (0.98–1.17)	**0.88** **(0.83–0.94)**	1.00 (0.94–1.07)	**0.91** **(0.86–0.97)**	0.97 (0.90–1.03)	**0.85** **(0.78–0.93)**	0.92 (0.83–1.01)

*CI* confidence interval, *cOR* crude odds ratio, *aOR* adjusted odds ratio, *Com* communication, *GM* gross motor, *FM* fine motor, *Prob* problem solving, *Perso* personal–social characteristics, *ASQ-3* Age and Stages Questionnaire, Third Edition.

Reference group: Participants without daily breakfast consumption habit.

Significant values are in bold.

Table 3 summarises the cORs, aORs, and 95% CIs for the outcomes of participants with DBH after stratification by maternal DEI and offspring sex. There were no significant differences in Group 1. In Group 2, the aORs were 0.80 (95% CI 0.68–0.94) and 0.66 (95% CI 0.47–0.95) for Com abnormalities among participants with DBH in both offspring sexes combined and only female offspring, respectively. The aOR was 0.67 (95% CI 0.45–0.99) for Perso abnormalities among participants with DBH in only female offspring. In Group 3, the aOR was 0.83 (95% CI 0.69–0.99) for Com abnormalities among participants with DBH in only male offspring. The aOR was 1.30 (95% CI 1.00–1.68) for GM abnormalities among participants with DBH in only female offspring. The aORs were 0.84 (95% CI 0.71–0.99) and 0.81 (95% CI 0.67–0.98) for Perso abnormalities among participants with DBH in both sexes combined and only male offspring, respectively.

**Table 3:** Crude and adjusted ORs for abnormalities in the ASQ-3 in participants with a daily breakfast consumption habit after stratification by maternal daily energy intake and offspring sex.

DEI Group^#^	Com	GM	FM	Prob	Perso
	aOR	aOR	aOR	aOR	aOR
1	0.89 (0.77–1.04)	1.01(0.88–1.17)	1.04 (0.94–1.17)	1.00 (0.89–1.12)	1.05 (0.89–1.25)
Male	0.92 (0.77–1.10)	0.94 (0.78–1.13)	1.06 (0.93–1.20)	0.99 (0.86–1.14)	1.02 (0.84–1.24)
Female	0.81 (0.60–1.10)	1.12 (0.89–1.41)	1.01 (0.81–1.27)	1.02 (0.84–1.24)	1.17 (0.80–1.69)
2	**0.80 (0.68–0.94)**	1.03 (0.88–1.21)	0.93 (0.83–1.06)	0.99 (0.87–1.12)	0.84 (0.70–1.01)
Male	0.83 (0.69–1.00)	1.07 (0.87–1.32)	0.94 (0.82–1.09)	1.02 (0.88–1.19)	0.89 (0.73–1.10)
Female	**0.66 (0.47–0.95)**	0.96 (0.75–1.24)	0.90 (0.70–1.14)	0.92 (0.73–1.15)	**0.67 (0.45–0.99)**
3	0.90 (0.77–1.05)	1.17 (0.99–1.37)	1.01 (0.90–1.13)	0.90 (0.80–1.01)	**0.84 (0.71–0.99)**
Male	**0.83 (0.69–0.99)**	1.09 (0.89–1.34)	1.01 (0.88–1.15)	0.88 (0.77–1.02)	**0.81 (0.67–0.98)**
Female	1.20 (0.87–1.65)	**1.30 (1.00–1.68)**	1.03 (0.82–1.29)	0.96 (0.78–1.17)	1.01 (0.70–1.48)

*CI* confidence interval, *cOR* crude odds ratio, *aOR* adjusted odds ratio, *Com* communication, *GM* gross motor, *FM* fine motor, *Prob* problem solving, *Perso* personal–social characteristics, *DEI* daily energy intake, *ASQ-3* Age and Stages Questionnaire, Third Edition.

^#^Group 1, less than 1400 kcal/day; Group 2, 1400–1800 kcal/day; Group 3, more than 1800 kcal/day.

Reference group: Participants without a daily breakfast consumption habit.

Significant values are in bold.

## Discussion

There is a paucity of reports on the association between DBH during pregnancy and offspring neurodevelopmental delays. The current study found that maternal DBH was associated with a significant reduction in Com and Perso abnormalities among offspring at 3 years of age, and this association changed based on maternal DEI and offspring sex. Particularly, there was no significant difference in neurodevelopmental delays at 3 years of age between offspring of participants with and without DBH during pregnancy in Group 1 (DEI < 1400 kcal). Nevertheless, Com and Perso abnormalities were significantly decreased in Groups 2 (DEI 1400–1799 kcal) and 3 (DEI ≥ 1800 kcal), respectively. Moreover, the incidence of GM abnormalities was significantly increased among female offspring in Group 3. To our best knowledge, this is the first nationwide cohort study reporting on this association with consideration of maternal DEI and offspring sex.

As hypothesised, there were significant reductions in Com and Perso abnormalities in Groups 2 and 3, which reflected the DEI of Japanese pregnant women in this study. Mato et al. reported that regular breakfast consumption was associated with a better nutritional balance in the diet, with more fruits and vegetables^13^. Additionally, Murakami et al. showed that breakfast accounted for 20–25% of DEI for those who consumed it. Compared with those who skipped breakfast, those who consumed breakfast had higher daily dietary quality scores as assessed using the Nutrient-Rich Food Index 9.3 (NRF9.3) and higher intakes of protein, n-6 and n-3 polyunsaturated fatty acids, carbohydrates, dietary fibre, vitamins, folic acid, calcium, magnesium, phosphorus, and iron^37^. In another study of middle school girls, those who consumed breakfast reported higher intakes of most nutrients (more vegetables and dairy product intake and less noodles and soft drink intake) than those who did not consume breakfast^38^. Thus, breakfast plays a key role in nutritional intake, and improving the quality of the mother’s diet with DBH will have a positive effect on the child’s neurodevelopment^39^.

Foetal brain growth and development are related to the various nutrients ingested by the mother; nutrients play a vital role in cell growth, DNA synthesis, neurotransmitter and hormone metabolism, and immune system development. Foetal brain development requires adequate energy and protein supplies, vitamins, essential fatty acids, and various key micronutrients^40,41^. Inadequate maternal nutrition during pregnancy interferes with placental cell proliferation and angiogenesis; thus, reducing nutrient supply to the foetus^42,43^. A deficiency of maternal vitamin D, which aids in neural differentiation and maturation and neurotransmitter synthesis, decreases the child’s mental and psychomotor development indices at 6 months^44^. Additionally, essential fatty acids, such as EPA and DHA, found in fish and other foods have a positive effect on neurodevelopment and immune function^45–47^. Hamazaki et al. reported that higher fish intake during pregnancy increased the child’s problem-solving ability at 6 months of age, FM skills, and problem-solving ability at 1 year of age^48^.

Intake of folic acid, an important nutrient for neurodevelopment, is recommended to prevent neural tube defect. Schlotz et al. reported that a lower folate status in early pregnancy might impair foetal brain development and cause hyperactivity, inattention, and peer problems in childhood^49^. Other micronutrients (e.g. zinc, iron, and iodine) also play important roles in foetal brain growth and development^50–53^. Previous findings support that DBH can improve intake of nutrients/quality of diet, thereby leading to better offspring neurodevelopment. It was unknown whether DBH in the current study population actually affected total daily nutrient and/or energy intake, as breakfast skippers could have compensated their intake at other meals. However, skipping breakfast has been found to lead to a lack of fatty acids, such as EPA and DHA, protein, and β-carotene, which are necessary for foetal growth^21^.

There was no significant difference in offspring neurodevelopmental delays at 3 years of age between those with and without DBH in Group 1. A DEI of < 1400 kcal/day is considerably below the dietary reference intake for Japanese pregnant women; therefore, this population is considerably undernourished^54^. Cortés-Albornoz et al. reported that inadequate nutrient intake during pregnancy was associated with reduced brain volume, brain defects (e.g. spina bifida), altered hypothalamic and hippocampal pathways, increased risk of abnormal behaviour, neuropsychiatric disorders (e.g. autism spectrum disorder and attention-deficit hyperactivity disorder), altered cognition, visual impairment, and motor deficits^4^. Regardless of DBH during pregnancy, participants in Group 1 would have considerable risks of offspring neurodevelopmental delay because low-energy intake itself has a strong effect on offspring neurodevelopment.

Contrary to our hypothesis, the incidence of GM abnormalities was significantly higher in female offspring of participants in Group 3. Although the underlying mechanism is unknown, there are some speculations. In the study by Graf et al., mice fed a high-fat diet experienced a neuroinflammatory response and decreased myelination in association with iron dysregulation in the foetal brain^55^. Other animal studies have also reported the possible effects of a high-fat diet on foetal neurodevelopment^56,57^. Thus, it is possible that DBH in the population of mothers with high DEIs might lead to unbalanced nutrient intake with high-fat content, which may affect foetal brain development and motor development. Further studies to clarify the effects of DBH during pregnancy in populations with higher DEIs are needed.

Moreover, sex-specific differences in neurodevelopmental effects of maternal DBH remain unexplored. However, the literature suggests that boys are more sensitive than girls and show a robust response to maternal inflammatory responses caused by maternal diet^55^. Furthermore, sex-specific differences have been reported with respect to postnatal effects of the intrauterine environment on mental illness, including schizophrenia^58^. Even in similar maternal environments, neurodevelopment may differ with offspring sex; however, this is a speculation warranting further research.

The current study findings emphasise that expectant mothers should be informed about the potential benefits of DBH and the effects of DEI during pregnancy. However, the DEI of the study population was below the required levels and did not meet the standard, with the mean DEI in the second half of pregnancy being 1735.63 kcal/day. Similarly, Kubota et al. reported that the mean DEI in any trimester of pregnancy in Japan was 1538–1595 kcal/day^59^, far below the recommended level of at least 1,900 kcal/day for those with low physical activities during the second half of pregnancy^60^. Therefore, both insufficient DEI and unhealthy dietary habits in pregnant women are major clinical and research issues in Japan. DBH may contribute to maternal DEI and may be a reliable marker of healthy dietary habits. Further studies to improve maternal diet with DBH and appropriate DEI leading to better offspring neurodevelopment are required.

This study has some limitations. First, the content and energy intake of the breakfast were not surveyed by the JECS group and were not included in this study. Second, the ASQ-3 was answered by caregivers, which may have led to recall bias. It is also expected that groups in which mothers consumed breakfast would be more likely to have their children consume breakfast as well. This could have influenced the results, but this was impossible to analyse. In addition, it is unclear how a decrease in Com abnormalities is specifically related to children’s neurodevelopment in the future. However, the study results may be useful in teaching pregnant women how to improve their nutritional status in the future. Moreover, there was a potential selection bias because of the exclusion of several participants owing to missing data and meeting the exclusion criteria. Finally, several unmeasured potential confounders may have affected the results; therefore, the results should be interpreted cautiously. Further research is needed to clarify the effects of DBH on perinatal health and the kind of food to be included in the breakfast, considering maternal DEI.

In conclusion, DBH during pregnancy has a negative effect on offspring neurodevelopmental delays, and this effect was dependent on maternal DEI and offspring sex. In Japan, low DEI among pregnant women is a longstanding issue; thus, comprehensive guidelines for a daily diet to improve offspring neurodevelopment are needed.

## Data Availability

Data are unsuitable for public deposition due to ethical restrictions and legal framework of Japan. It is prohibited by the Act on the Protection of Personal Information (Act No. 57 of 30 May 2003, amendment on 9 September 2015) to publicly deposit the data containing personal information. Ethical Guidelines for Epidemiological Research enforced by the Japan Ministry of Education, Culture, Sports, Science and Technology and the Ministry of Health, Labour and Welfare also restrict the open sharing of the epidemiologic data. All inquiries about access to data should be sent to: jecs-en@nies.go.jp. The person responsible for handling enquiries sent to this e-mail address is Dr Shoji F. Nakayama, JECS Programme Office, National Institute for Environmental Studies.

## Abbreviations

aOR: Adjusted odds ratio
AQ: Autism spectrum quotient
ART: Assisted reproductive technology
ASQ-3: Ages and Stages Questionnaire, Third Edition
BMI: Body mass index
CI: Confidence interval
Com: Communication
DBH: Daily breakfast consumption habit
cORs: Crude odds ratios
DEI: Daily energy intake
DHA: Docosahexaenoic acid
EPA: Eicosapentaenoic acid
FM: Fine motor
GM: Gross motor
JECS: Japan Environment and Children’s Study
JPY: Japanese yen
K6: Kessler 6
Perso: Personal–social characteristics
Prob: Problem solving
